# Toward a Cytological Classification of Nasal Biofilms in Upper Airway Inflammation: Microscopic Patterns, Clinical Meaning, and Therapeutic Implications

**DOI:** 10.3390/biomedicines14092076

**Published:** 2026-09-15

**Authors:** Matteo Gelardi

**Affiliations:** Unit of Otolaryngology, Department of Clinical and Experimental Medicine, University of Foggia, Viale Pinto, 1, 71122 Foggia, Italy; matteo.gelardi@unifg.it

**Keywords:** nasal cytology, nasal biofilm, upper airway inflammation, chronic rhinosinusitis, cytological classification, infectious spot, fungal elements, precision medicine

## Abstract

**Background/Objectives:** Nasal biofilms are increasingly recognized as contributors to chronic and recurrent upper airway inflammation, yet their cytological interpretation is still often reduced to a binary presence/absence judgment, underestimating their morphological, microbial, and inflammatory heterogeneity. This Perspective proposes a cytological classification framework for nasal biofilms based on microscopic morphology, degree of organization, bacterial and fungal composition, host inflammatory response, and therapeutic implications. **Methods:** This conceptual framework integrates standardized nasal cytology, microscopic observation of bacterial aggregates and biofilm-like structures, May–Grünwald–Giemsa staining features, and current knowledge of biofilm behavior in upper airway inflammatory disorders, focusing on relationships among bacteria, fungal elements, mucus, epithelial cells, and inflammatory infiltrates. **Results:** Five cytological patterns are proposed: free bacterial colonization, microaggregate biofilm-like pattern, structured/mature biofilm pattern, inflammatory biofilm-associated pattern, and dispersal/disrupted biofilm pattern, reflecting different degrees of microbial organization and host–microbe interaction and possibly coexisting within the same smear. Nasal cytology may also reveal fungal-predominant or mixed bacterial–fungal patterns, supporting an “infectious condominium” concept, and should distinguish isolated fungal spores from the rarer, clinically more significant vegetative forms such as hyphae. **Conclusions:** Moving from a presence/absence approach to pattern-based interpretation may improve the diagnostic and clinical value of nasal cytology. Although prospective validation is required, this classification offers a shared descriptive language that may support more precise phenotyping of upper airway inflammation, avoid overinterpretation of simple colonization, and inform personalized diagnostic and therapeutic strategies.

## 1. Introduction

Biofilms represent one of the most successful and persistent forms of microbial life. Rather than existing as isolated planktonic cells, bacteria within biofilms organize into structured communities embedded in a self-produced extracellular polymeric substance matrix, which provides mechanical stability, protection from environmental stressors, and increased tolerance to antimicrobial agents [1,2,3]. This organization is dynamic rather than static: biofilms undergo adhesion, microcolony formation, maturation, detachment, and dispersion, behaving as complex microbial systems rather than simple bacterial aggregates [4,5,6,7].

In human disease, biofilms have been widely implicated in chronic and recurrent infections, particularly when mucosal surfaces are involved [8]. In the upper airways, the sinonasal mucosa provides a favorable ecological niche where mucus, epithelial cells, microbial communities, inflammatory mediators, and environmental exposures interact continuously. Within this context, nasal and sinonasal biofilms have been associated with chronic rhinosinusitis, recurrent inflammatory disease, impaired mucociliary clearance, persistence of symptoms, and reduced response to conventional therapies [9,10].

The current understanding of upper airway inflammation has progressively shifted from a pathogen-centered view to an ecosystem-based model, in which the nasal and nasopharyngeal microbiome is considered an active regulator of mucosal homeostasis and immune response rather than a passive microbial background. Dysbiosis, loss of microbial diversity, expansion of potentially pathogenic taxa, and biofilm formation may contribute to chronic inflammatory ear, nose, and throat diseases, including chronic rhinosinusitis and allergic airway disorders [11,12]; this is particularly relevant in chronic rhinosinusitis, where microbial imbalance and biofilm organization may interact with epithelial barrier dysfunction, mucociliary impairment, and local inflammatory patterns.

Despite advances in microbiome sequencing, confocal microscopy, fluorescence in situ hybridization, scanning electron microscopy, and culture-independent techniques, many of these approaches remain complex, expensive, or poorly suited to routine clinical monitoring. Nasal cytology, by contrast, offers a simple, repeatable, minimally invasive, and direct microscopic assessment of the nasal mucosa: sampling by scraping of the inferior turbinate, followed by May–Grünwald–Giemsa staining, allows the simultaneous evaluation of epithelial cells, inflammatory infiltrates, mucus, bacteria, fungal elements, and biofilm-like structures within the same microscopic field [13,14,15,16,17]. This integrated perspective does not isolate the microbial component from the host inflammatory context.

Previous cytological studies have described the so-called “infectious spot” as a morphological-chromatic expression of nasal biofilm and have shown that nasal cytology can detect biofilm-like structures in different forms of rhinitis and sinonasal inflammatory disease [13,14,16,17]. More recently, nasal cytology has gained renewed relevance as a tool for inflammatory phenotyping and precision medicine in respiratory diseases [18], and its methodological standardization has been strengthened by expert-based consensus initiatives supporting its reproducibility and clinical applicability [19].

One major limitation remains, however: in both clinical practice and scientific reporting, nasal biofilm is often interpreted in a binary manner, as simply present or absent. Free bacteria, small bacterial aggregates, structured biofilm-like formations, inflammatory biofilm-associated patterns, and disrupted or dispersing biofilms may represent different biological and clinical conditions; the presence of bacteria is not equivalent to the presence of a structured biofilm, and the presence of a biofilm-like structure may carry different implications depending on its degree of organization and associated inflammatory pattern.

For this reason, a cytological classification of nasal biofilms may be useful, not to replace microbiological, molecular, or ultrastructural methods, but to provide a practical interpretative framework for routine microscopy. Moving from a simple presence/absence assessment toward a pattern-based cytological interpretation may help distinguish bacterial colonization from structured biofilm, identify biofilm-associated inflammation, and support more personalized therapeutic strategies.

This Perspective proposes a cytological classification of nasal biofilms based on microscopic morphology, degree of organization, relationship with mucus and epithelial cells, inflammatory context, and possible dispersal features, aiming to provide a conceptual and practical framework for interpreting nasal biofilms in upper airway inflammation, with potential diagnostic, prognostic, and therapeutic implications.

## 2. Rationale for a Cytological Classification of Nasal Biofilms

The identification of nasal biofilms has traditionally been approached as a qualitative finding, based mainly on presence or absence. Although useful as an initial diagnostic step, this binary interpretation does not fully reflect the morphological complexity of biofilm-related findings observed in nasal cytology. Biofilms are dynamic microbial communities characterized by matrix production, structural organization, maturation, and dispersion; their microscopic appearance may therefore vary according to their biological state [1,2,3,4,5,6,7]. In routine cytological evaluation, bacterial elements may appear as isolated free bacteria, small aggregates, compact biofilm-like structures, biofilm-associated inflammatory clusters, or fragmented/dispersing formations, appearances that are not necessarily equivalent and may correspond to different biological conditions.

From a cytological perspective, the distinction between bacterial colonization and structured biofilm is particularly relevant. Free bacteria dispersed in mucus may indicate simple colonization, contamination, or transient bacterial overgrowth, whereas compact bacterial aggregates embedded in a matrix-like material and closely associated with mucus, epithelial cells, or inflammatory elements may suggest a more organized microbial community [13,14,15,16,17]. The microscopic organization of bacteria, rather than their mere presence, should therefore be considered a key interpretative criterion.

Another relevant aspect is the relationship between biofilm-like structures and the inflammatory microenvironment. Nasal cytology allows direct visualization of the cellular context in which bacterial aggregates are located, including epithelial cells, neutrophils, eosinophils, mast cells, mucus, and degenerative cellular material [15,18,19]. A biofilm-like structure surrounded by neutrophils may suggest a predominantly infectious or innate inflammatory response, whereas coexistence with eosinophils, mast cells, dense mucus, epithelial damage, or Charcot–Leyden crystals may indicate a more complex inflammatory interaction, particularly relevant in chronic rhinosinusitis and other persistent upper airway disorders, where microbial organization, dysbiosis, epithelial dysfunction, and host inflammation may mutually reinforce each other [9,10,11,12].

The degree of structural organization may also carry potential clinical implications. Poorly organized microaggregates may represent an early or unstable phase, while dense, compact formations may be more consistent with mature biofilm; disrupted structures with peripheral bacterial satellites may suggest biofilm fragmentation or dispersal, in line with the dynamic nature of the biofilm life cycle [4,5,6,7]. Although cytology cannot replace molecular or ultrastructural methods, it can provide a practical morphological framework for interpreting these patterns in daily clinical practice [13,14,15,16,17].

A cytological classification may therefore help standardize the descriptive language used for nasal biofilms. Instead of reporting only “biofilm present” or “biofilm absent,” the cytological report could identify a specific pattern, integrating bacterial morphology, matrix-like appearance, mucus relationship, epithelial involvement, inflammatory background, and possible dispersal features, consistent with the broader need for standardized nasal cytology methodology and terminology recently reinforced by expert consensus initiatives [19].

The aim of the proposed classification is not to establish a definitive or universally validated staging system, but to provide a conceptual and practical framework that can be tested in future studies. A pattern-based approach may help generate new hypotheses regarding the relationship between biofilm morphology, inflammatory phenotype, disease persistence, therapeutic response, and recurrence risk, contributing not only to diagnosis but also to precision medicine in upper airway inflammation [18,19].

## 3. Practical Cytological Assessment of Nasal Biofilms

Reliable application of the proposed classification requires standardized sampling, slide preparation, staining, and microscopic interpretation. Nasal cytology is generally performed by collecting superficial mucosal material, commonly from the inferior turbinate, followed by smear preparation and May–Grünwald–Giemsa staining according to established procedures [13,14,15,16,17]. Because the present classification does not require a specific or modified sampling technique, detailed methodological aspects are not repeated here.

For biofilm assessment, particular attention should be directed to bacterial distribution and aggregation, matrix-like material, chromatic characteristics, relationship with mucus and epithelial elements, associated inflammatory cells, fungal elements when present, and signs of fragmentation or disruption. Interpretation should be performed at different magnifications and within the overall cytological context rather than on the basis of a single microscopic feature.

Fungal elements, when identified, should be described separately from bacterial biofilm-like findings, specifying whether spores, hyphae, or mixed fungal–bacterial structures are present. Spores are generally more frequent and less specific, whereas hyphae are less commonly observed but may carry greater clinical relevance and therefore require careful clinical correlation. Bacterial and fungal elements may coexist within the same biofilm-like matrix. Cytology may support morphological characterization of these findings but does not provide definitive species identification or establish tissue invasion.

Standardization of sample collection, smear preparation, staining quality, and interpretative criteria is therefore a prerequisite for reproducible application of the proposed framework.

**Figure 1:**
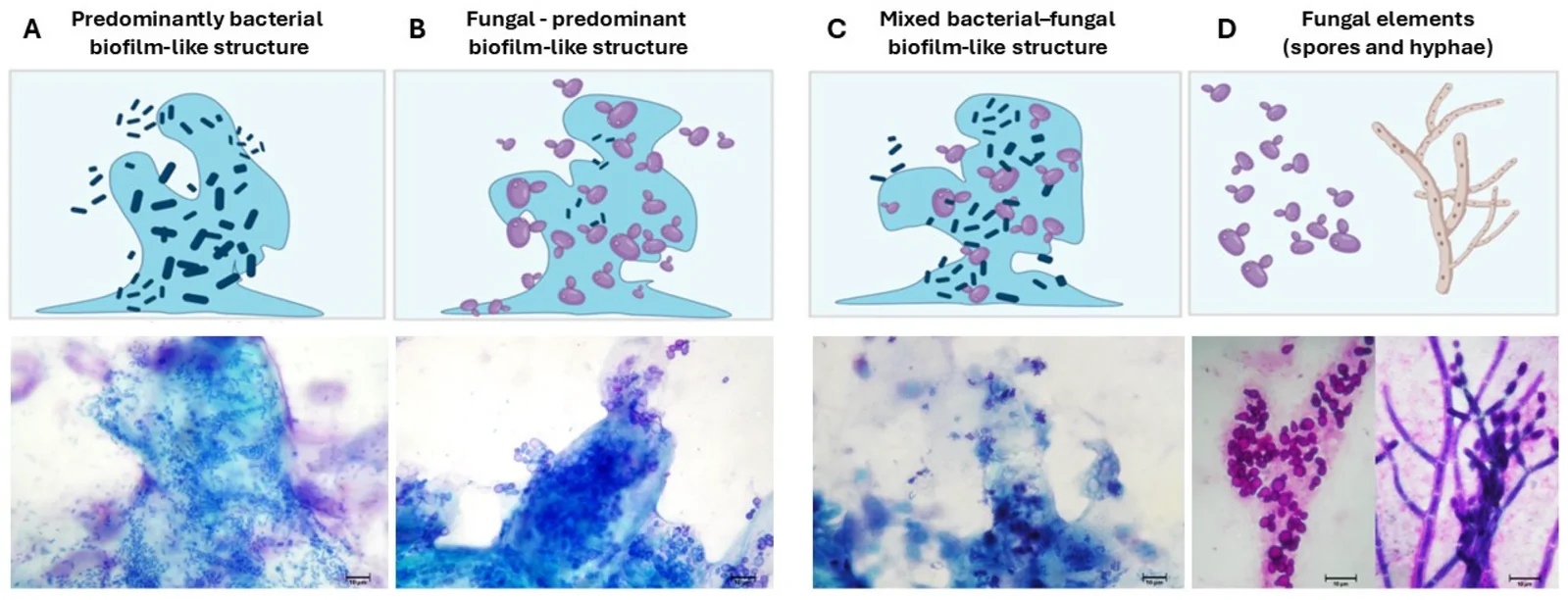
Cytological microbial composition of nasal biofilm-like structures. (**A**) Predominantly bacterial biofilm-like structure. (**B**) Fungal-predominant biofilm-like structure. (**C**) Mixed bacterial–fungal biofilm-like structure, illustrating the coexistence of bacterial and fungal elements within the same matrix-like material. (**D**) Fungal elements identifiable in nasal cytology, including spores and hyphae. In each panel, the schematic representation is paired with a representative real nasal cytology microphotograph to facilitate morphological recognition. Scale bars: 10 μm. Schematic illustrations were created with BioRender.com. Gelardi, M. (2026), https://BioRender.com/z3y9eah, accessed on 13 September 2026. All cytological microphotographs are representative microscopic findings selected from the author’s teaching archive for illustrative purposes only. The images are fully anonymized and contain no patient-identifying information. They are included solely to illustrate the morphological findings discussed in this Perspective and do not represent a new patient cohort or a new analysis of clinical data.

The main cytological features considered in the assessment of bacterial, fungal, and mixed microbial patterns are summarized in Figure 1.

### Differential Diagnosis and Diagnostic Pitfalls

Cytological recognition of biofilm-like structures requires careful distinction from non-specific aggregates and technical artefacts. Mucus clumps may produce dense, irregular, or intensely stained structures that can superficially resemble matrix-like material, particularly in thick smears. A true biofilm-like interpretation should therefore rely not on mucus density or staining intensity alone, but on the recognition of organized bacterial aggregates within a compatible matrix-like and cellular context.

Epithelial debris and degenerating cellular material may also mimic irregular biofilm-like formations. Their cellular origin can usually be suggested by residual nuclear or cytoplasmic features and by the absence of a coherent bacterial organization. Similarly, staining precipitates, excessive stain deposition, uneven May–Grünwald–Giemsa coloration, or poorly prepared slides may generate granular or amorphous structures that should not be interpreted as microbial aggregates.

Contamination represents another potential source of misinterpretation. Scattered bacteria introduced during sampling, slide preparation, or handling may be difficult to distinguish from superficial colonization when considered in isolation. Interpretation should therefore take into account bacterial distribution, structural organization, relationship with mucus and epithelial elements, and the associated inflammatory background rather than bacterial presence alone.

Recent antibiotic or topical antimicrobial treatment may reduce bacterial density or modify the morphology of microbial aggregates, potentially producing incomplete, fragmented, or poorly organized appearances. Likewise, nasal irrigation, mucolytic treatment, and other local therapies may alter mucus architecture and mechanically disrupt biofilm-like material. Treatment history and timing of sampling should therefore be considered when assigning a cytological pattern.

Finally, mechanical factors related to sampling and smear preparation may themselves generate fragmentation. Scraping pressure, transfer of material to the slide, and spreading of viscous mucus may disrupt compact aggregates and create an appearance resembling the proposed dispersal/disrupted pattern. For this reason, Pattern V requires particularly cautious interpretation and should not be considered evidence of biological biofilm dispersion in the absence of appropriate clinical context or longitudinal confirmation.

**Figure 2:**
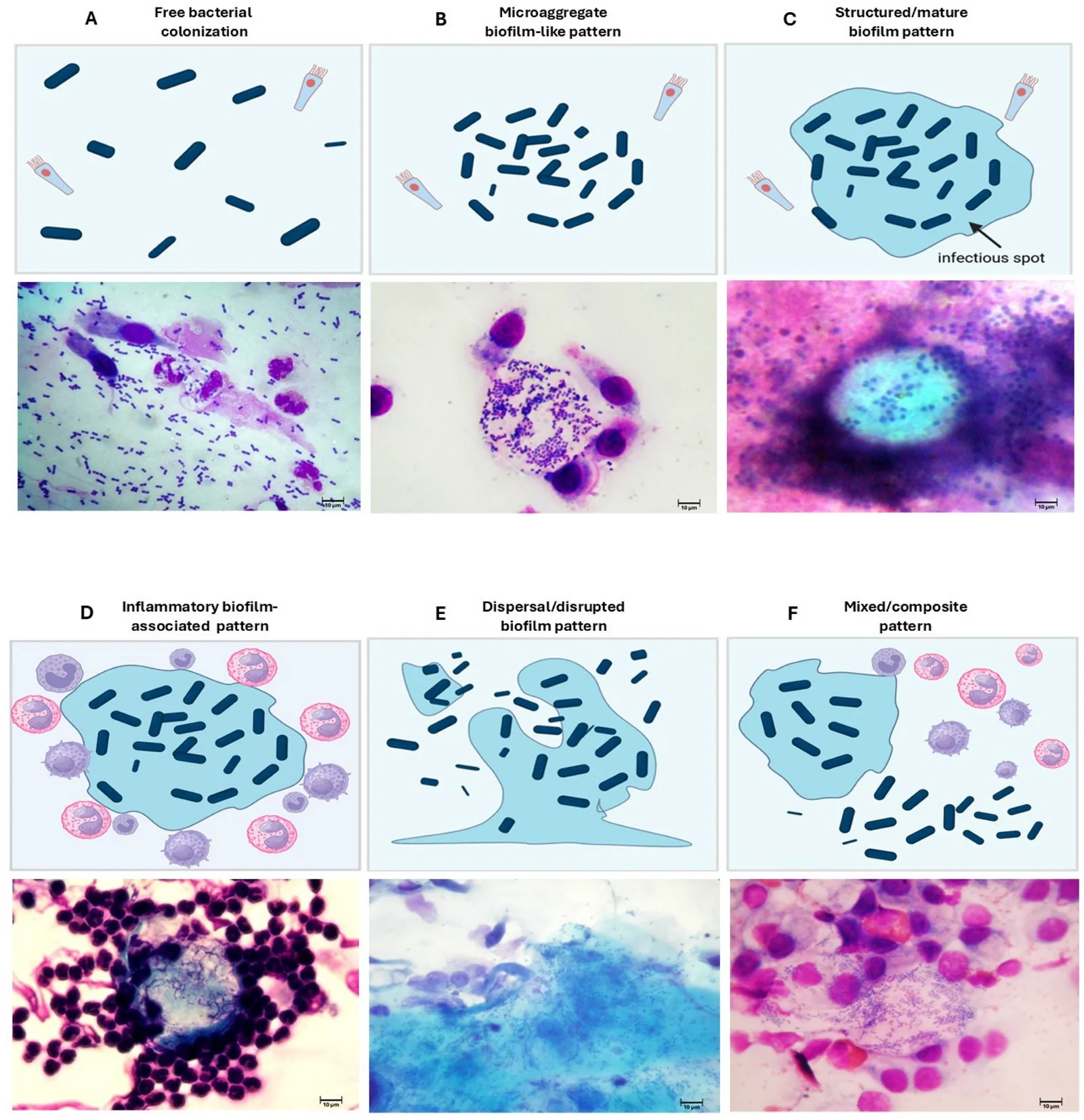
Proposed cytological patterns of nasal biofilms. (**A**) Free bacterial colonization, characterized by scattered bacterial elements without recognizable matrix-like organization. (**B**) Microaggregate biofilm-like pattern, showing small bacterial clusters with early aggregation and limited matrix-like material. (**C**) Structured/mature biofilm pattern, characterized by compact bacterial aggregates embedded in cyanophilic matrix-like material, corresponding morphologically to the previously described “infectious spot”. (**D**) Inflammatory biofilm-associated pattern, showing organized biofilm-like material within a prominent inflammatory-cell background. (**E**) Dispersal/disrupted biofilm pattern, characterized by fragmented matrix-like material and peripheral bacterial release. (**F**) Mixed/composite pattern, illustrating coexistence of more than one cytological pattern within the same sample. In each panel, the schematic representation is paired with a representative real nasal cytology microphotograph to facilitate morphological recognition. The proposed patterns should be interpreted as components of a cytological continuum rather than as mutually exclusive categories. Scale bars: 10 μm. Schematic illustrations were created with BioRender.com. Gelardi, M. (2026), https://biorender.com/z3y9eah, accessed on 13 September 2026. All cytological microphotographs are representative microscopic findings selected from the author’s teaching archive for illustrative purposes only. The images are fully anonymized and contain no patient-identifying information. They are included solely to illustrate the morphological findings discussed in this Perspective and do not represent a new patient cohort or a new analysis of clinical data.

Overall, reliable interpretation should integrate bacterial organization, matrix-like appearance, chromatic features, epithelial and inflammatory context, treatment history, and technical quality of the preparation. No single morphological or chromatic feature should be considered diagnostic of biofilm in isolation. The cytological report should therefore describe not only the presence of biofilm-like material, but also its morphology, chromatic pattern, degree of organization, associated inflammatory background, and possible signs of disruption or dispersal. The proposed classification extends this ongoing standardization effort in nasal cytology, applying the same need for reproducible terminology and shared interpretative criteria to biofilm-related findings; the proposed cytological patterns are illustrated in Figure 2 through paired schematic representations and representative cytological microphotographs.

## 4. Proposed Cytological Classification of Nasal Biofilms

The proposed classification is based on the microscopic appearance of bacterial elements, their degree of organization, their relationship with mucus and epithelial cells, the associated inflammatory background, and the presence of possible disruption or dispersal features. It should be read as a practical morphological framework rather than a validated staging system, moving cytological interpretation beyond a binary presence/absence model and toward a pattern-based reading of biofilm organization and host–microbe interaction.

To improve descriptive reproducibility, each pattern is defined using a limited set of morphological domains: bacterial distribution and aggregation, apparent structural compactness, presence and appearance of matrix-like material, relationship with epithelial cells and mucus, associated inflammatory background, and evidence of fragmentation or peripheral bacterial release. At present, validated quantitative cut-offs for aggregate size, bacterial density, matrix extent, or inflammatory-cell counts are not available for nasal cytology. Therefore, the proposed criteria are deliberately morphological and semi-quantitative rather than numerical, and future validation studies should determine whether reproducible dimensional or cellular thresholds can be established.

Five main cytological patterns may be recognized: free bacterial colonization, microaggregate biofilm-like pattern, structured/mature biofilm pattern, inflammatory biofilm-associated pattern, and dispersal/disrupted biofilm pattern. Representative real cytological microphotographs corresponding to each proposed pattern are provided in Figure 2 to complement the schematic illustrations and facilitate morphological recognition. Representative fungal-predominant and mixed bacterial–fungal findings are illustrated in Figure 1. These patterns may coexist in the same sample, particularly in chronic or recurrent inflammatory conditions, and should be interpreted within the broader cytological and clinical context.

### 4.1. Pattern I: Free Bacterial Colonization

This pattern is characterized by free or scattered bacterial elements within the mucus, without a clear matrix-like background or organized aggregation. Bacteria may appear as isolated cocci, bacilli, or mixed forms, sometimes associated with mucus strands or superficial epithelial debris, without compact clustering, three-dimensional organization, or a recognizable biofilm-like architecture.

This finding should not automatically be interpreted as structured biofilm: it may represent transient colonization, contamination, superficial bacterial overgrowth, or a nonspecific microbial presence within nasal secretions, and should be distinguished from true biofilm-like formations, in which bacterial aggregation and matrix-associated organization are more evident [1,2,3,13,14,15,16,17].

Its possible clinical meaning is limited if considered alone, and increases when associated with neutrophilic inflammation, epithelial damage, abundant mucus, or symptoms suggestive of infectious exacerbation. In the absence of structural organization or a significant inflammatory reaction, this pattern should be interpreted cautiously and should not necessarily lead to antimicrobial treatment.

### 4.2. Pattern II: Microaggregate Biofilm-like Pattern

This pattern is characterized by small bacterial clusters or microaggregates, often embedded in mucus and sometimes adjacent to epithelial cells. Compared with free colonization, bacteria show an initial tendency to aggregate, but the structure remains incomplete, poorly defined, and lacks the compact architecture typical of mature biofilm.

This pattern may represent an intermediate condition between simple bacterial colonization and structured biofilm. Early biofilm formation involves bacterial adhesion, microcolony development, and progressive matrix production [4,5]; however, cytology alone cannot definitively establish the developmental stage of a biofilm, so the term “biofilm-like” is appropriate when the morphology suggests early organization but lacks the features of a mature, compact structure.

Clinically, Pattern II may indicate a potentially unstable or early phase of microbial organization, with its significance depending on persistence over repeated cytological evaluations, association with symptoms or inflammatory infiltrate, impaired mucociliary function, or chronicity of the underlying disease.

### 4.3. Pattern III: Structured/Mature Biofilm Pattern

This pattern is characterized by compact, dense, and organized bacterial aggregates embedded in amorphous matrix-like material, with a cohesive appearance, irregular borders, and a three-dimensional cytological architecture, often closely associated with mucus, epithelial cells, or cellular debris. Compared with Pattern II, bacterial organization is more evident and the structure appears more stable and consolidated, representing the cytological expression most consistent with structured or mature biofilm, which is characterized by extracellular polymeric substance production, microbial community organization, and increased tolerance to environmental stressors and antimicrobial agents [1,2,3,4,7,8]. In nasal cytology, this organization may be suggested by dense bacterial aggregates with matrix-like material and the characteristic morphological-chromatic appearance previously described as the “infectious spot” [13,16,17].

Its clinical relevance may be greater than that of simple colonization or microaggregation: structured biofilm may contribute to persistence of inflammation, chronicity of symptoms, impaired response to conventional treatment, and recurrence in selected patients with chronic rhinitis or chronic rhinosinusitis [9,10,17]. However, cytological evidence of structured biofilm should always be integrated with clinical presentation, endoscopic findings, inflammatory pattern, and, when appropriate, microbiological or molecular investigations.

### 4.4. Pattern IV: Inflammatory Biofilm-Associated Pattern

This pattern is characterized by the coexistence of biofilm-like structures and a significant inflammatory background. The bacterial aggregate may be surrounded by or intermingled with neutrophils, eosinophils, mast cells, macrophages, epithelial debris, dense mucus, or degenerative material, and Charcot–Leyden crystals may occasionally be observed, suggesting eosinophilic inflammation.

This pattern is particularly relevant because it shifts the interpretation from a purely microbiological finding to a host–microbe interaction. A biofilm-like structure associated with neutrophils may suggest an infectious or innate immune response, whereas association with eosinophils and mast cells may indicate a more complex inflammatory environment, especially in type 2 inflammatory disorders of the upper airways. Nasal cytology is uniquely positioned to identify this relationship because it allows simultaneous visualization of bacteria, mucus, epithelial damage, and inflammatory cells within the same microscopic field [15,18,19].

In chronic rhinosinusitis and other persistent upper airway inflammatory diseases, biofilm, dysbiosis, epithelial barrier dysfunction, and inflammatory persistence may act together, amplifying disease chronicity and possibly influencing therapeutic response [9,10,11,12]. Pattern IV may therefore represent the most clinically informative cytological category, describing not merely biofilm presence but the inflammatory context in which it is embedded.

### 4.5. Pattern V: Dispersal/Disrupted Biofilm Pattern

This pattern is characterized by fragmented biofilm-like material, irregular disruption of previously compact aggregates, peripheral bacterial satellites, or apparent release of bacterial elements from a matrix-like structure, with partially disorganized clusters, small detached fragments, and scattered bacteria surrounding a residual compact core.

This pattern is consistent with the dynamic concept of biofilm detachment or dispersion, a recognized phase of the biofilm life cycle that may occur through active bacterial release, matrix degradation, mechanical disruption, or environmental stress [5,6,7]. In cytology, however, interpretation must remain cautious, because a disrupted appearance may also result from sampling, smear preparation, mucus fragmentation, or treatment effects.

Its clinical implications may be important but require validation: dispersal may contribute to bacterial spreading, recurrence, exacerbation, or recolonization of adjacent mucosal sites, but a disrupted pattern may equally be observed after local treatment, nasal irrigation, mucolytic strategies, or partial biofilm destabilization. Pattern V should therefore be interpreted in relation to timing of sampling, ongoing therapy, symptom dynamics, and comparison with previous cytological findings.

### 4.6. Mixed and Composite Cytological Biofilm Patterns

In routine nasal cytology, the proposed patterns should not be interpreted as mutually exclusive categories. A single smear may contain more than one biofilm-related pattern, particularly in chronic or recurrent upper airway inflammation, reflecting spatial heterogeneity of the nasal mucosal surface, different stages of microbial organization, variable interactions with mucus and epithelial cells, or dynamic processes such as partial disruption and dispersal.

Cytological reporting should therefore identify the dominant pattern, defined as the most representative or clinically relevant finding, together with any associated secondary patterns that provide additional information on organization, inflammatory interaction, or dispersal. For example, coexistence of free bacterial colonization and microaggregates may suggest early microbial organization, while coexistence of structured biofilm and inflammatory biofilm-associated features may indicate a more clinically relevant host–microbe interaction. Similarly, the simultaneous presence of structured/mature biofilm and disrupted/dispersal features may suggest instability, partial fragmentation, or active bacterial release.

The proposed classification should therefore be applied as a flexible interpretative framework rather than a rigid categorical system, for example, “dominant structured/mature biofilm pattern with associated inflammatory biofilm features” or “microaggregate biofilm-like pattern with focal dispersal/disrupted areas.” This approach may better reflect the real microscopic complexity of nasal biofilms and improve communication between cytologists and clinicians.

### 4.7. Summary of the Proposed Cytological Patterns

The five proposed patterns should be considered components of a cytological continuum rather than rigid diagnostic categories. In clinical practice, the final interpretation should include the dominant pattern, associated secondary patterns, and the inflammatory background in which they are observed; their main value lies in improving the descriptive precision of nasal cytology and in generating hypotheses for future clinical validation. Table 1 summarizes the five proposed patterns, their main microscopic features, and their tentative clinical interpretation.

The evidentiary basis of the proposed patterns is not uniform. Some components, particularly the structured/mature biofilm pattern corresponding to the previously described “infectious spot”, are directly supported by published nasal cytology observations. Other patterns are derived from the integration of established biofilm biology with recurring microscopic appearances observed in routine nasal cytology. In particular, the microaggregate and dispersal/disrupted patterns should presently be regarded as hypothesis-generating cytological interpretations rather than independently validated biological stages. Table 2 summarizes the level and nature of evidence supporting each proposed pattern and explicitly distinguishes published observations from the author’s interpretative contribution.

The proposed patterns are not intended as purely theoretical constructs. They integrate recurrent cytological appearances described in published nasal cytology studies with established biological features of biofilm development and with observations reported using complementary microscopic and molecular techniques. The degree of direct clinical and cytological support differs among patterns and is summarized in Table 2.

## 5. Clinical Interpretation of Cytological Biofilm Patterns

The clinical value of the proposed classification lies in interpreting nasal biofilms not as isolated microscopic findings, but as part of a broader cytological and clinical picture. The same biofilm-like structure may carry different significance depending on its degree of organization, inflammatory background, persistence over time, and association with symptoms, endoscopic findings, mucociliary impairment, or recurrent disease. Cytological biofilm patterns should therefore always be interpreted together with the patient’s clinical phenotype, not as stand-alone diagnostic categories.

A critical point is that the microscopic detection of bacteria, fungal spores, hyphae, mixed bacterial–fungal elements, or biofilm-like structures does not automatically imply pathogenic infection. Nasal cytology provides morphological and contextual information but does not identify microbial species, virulence factors, antimicrobial resistance profiles, or the functional behavior of the microbial community. The proposed classification should therefore be understood as a morphological and inflammatory framework, not as a microbiological diagnosis of pathogenicity.

The nasal mucosa normally harbors complex microbial communities that include commensal, saprophytic, opportunistic, and potentially pathogenic organisms. Bacteria observed on a cytological smear, including low-virulence commensals such as viridans group streptococci, may belong to resident or transient microbiota rather than to true pathogens. Their presence, in the absence of epithelial damage, neutrophilic inflammation, dense pathological mucus, recurrent symptoms, or treatment failure, should be interpreted cautiously. The same principle applies to fungal elements: isolated spores should not be equated with fungal disease, as they may reflect environmental exposure, transient colonization, or saprophytic presence, whereas vegetative forms, particularly septate or broad pauciseptate/non-septate hyphae, change the level of clinical concern and, when associated with necrotic debris, epithelial damage, severe neutrophilic inflammation, immunosuppression, or poorly controlled diabetes, should prompt further clinical, endoscopic, radiological, microbiological, mycological, and histopathological correlation [24,25,26].

Read together with the pattern definitions in Section 4, this framework stratifies cytological findings by increasing clinical relevance: free bacterial colonization (Pattern I) is the least specific finding and should not, by itself, be treated as clinically significant biofilm; microaggregate and structured patterns (Patterns II–III) reflect increasing degrees of microbial organization and gain importance when persistent or associated with chronic symptoms; the inflammatory biofilm-associated pattern (Pattern IV) is arguably the most clinically informative, because it characterizes the host–microbe interaction rather than the microbial structure alone; and the dispersal/disrupted pattern (Pattern V) requires the most cautious interpretation, since its dynamic and heterogeneous appearance may reflect active biofilm behavior, disease exacerbation, or artefacts of treatment and sample handling.

This classification may also improve communication between cytologists and clinicians. Instead of reporting only “biofilm present,” a cytological report could describe the finding as, for example, “dominant structured/mature biofilm pattern with associated neutrophilic inflammation,” “microaggregate biofilm-like pattern without significant inflammatory background,” “inflammatory biofilm-associated pattern with eosinophil–mast cell infiltration,” or “mixed bacterial–fungal microbial context with vegetative fungal forms requiring clinical correlation.” Such terminology may better reflect the biological complexity of the smear and support more individualized clinical interpretation.

**Figure 3:**
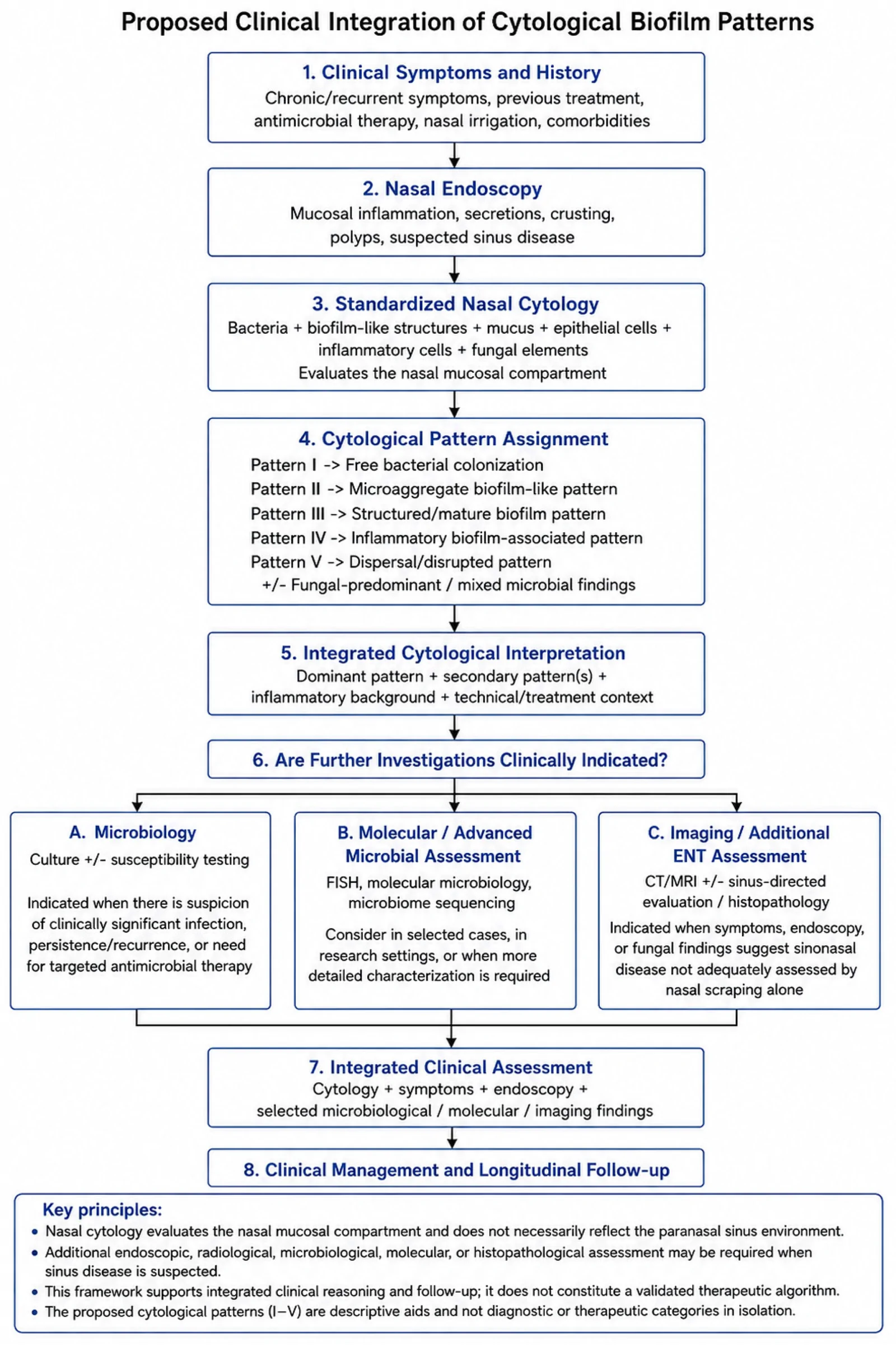
Proposed clinical integration of cytological biofilm patterns. Nasal cytological findings should be interpreted within the clinical and endoscopic context rather than as stand-alone diagnostic categories. Following standardized nasal cytology, the dominant biofilm-related pattern, associated secondary patterns, inflammatory background, fungal findings, and relevant technical or treatment-related factors are integrated with symptoms and nasal endoscopy. Microbiological culture, molecular approaches, and imaging or additional sinonasal investigations are selectively considered according to clinical presentation, persistence or recurrence, discordant findings, and suspicion of sinus or fungal disease. The framework is intended to support integrated clinical reasoning and longitudinal assessment and does not constitute a validated diagnostic or therapeutic algorithm.

To facilitate translation of the proposed classification into clinical practice, cytological findings should be interpreted within a stepwise multidisciplinary framework rather than in isolation. As summarized in Figure 3, clinical symptoms and history provide the initial context, followed by nasal endoscopy and nasal cytology. The cytological pattern should then be integrated with the inflammatory background and endoscopic findings. Microbiological culture, molecular testing, and imaging should be considered selectively when required by disease severity, persistence, recurrence, discordant findings, or suspicion of sinonasal or fungal disease. The resulting integrated assessment may support clinical interpretation and follow-up, while avoiding the use of cytological biofilm patterns as stand-alone diagnostic or therapeutic criteria.

An important limitation of routine nasal cytology is that samples obtained from the inferior turbinate reflect the nasal mucosal compartment and should not be assumed to represent the microbiological or structural organization of biofilms within the paranasal sinuses. In patients with chronic rhinosinusitis, and particularly when fungal sinus disease or fungal ball is suspected, sinonasal compartments may differ substantially in microbial composition and biofilm architecture. In such cases, cytological findings should be integrated with nasal endoscopy, radiological imaging, and, when clinically indicated, sinus-directed microbiological or histopathological assessment.

## 6. Therapeutic Implications

The proposed cytological classification is not intended to function as a direct therapeutic algorithm. Rather, its potential value lies in supporting more nuanced clinical reasoning, helping clinicians distinguish simple microbial detection from structured biofilm organization, biofilm-associated inflammation, and dynamic or mixed microbial patterns. Nasal cytology may thereby contribute to therapeutic decision-making by providing information on the morphology of microbial aggregates, their inflammatory context, and their persistence or modification over time.

A first practical implication is the need to avoid overtreatment. Detection of free bacteria (Pattern I) should not automatically lead to antimicrobial therapy: scattered bacterial elements may reflect colonization, contamination, or transient microbial overgrowth, especially in the absence of epithelial damage, neutrophilic inflammation, dense pathological mucus, or recurrent symptoms. Here, cytology may help prevent inappropriate antibiotic use by distinguishing simple bacterial presence from clinically relevant biofilm-associated inflammation.

Pattern II, the microaggregate biofilm-like pattern, suggests an early or unstable degree of microbial organization and should be interpreted cautiously; in many cases it may justify clinical and cytological follow-up rather than immediate antimicrobial intervention, with repeated nasal cytology helping determine whether microaggregates disappear, persist, evolve toward structured biofilm, or become associated with inflammatory activation. General measures aimed at improving nasal hygiene, mucus clearance, and local mucosal conditions may be more appropriate than empiric systemic therapy.

Pattern III, the structured/mature biofilm pattern, may have greater therapeutic relevance because it suggests a more organized microbial community; mature biofilms show increased tolerance to antimicrobial agents and environmental stressors, mainly because of their extracellular matrix, spatial organization, and altered microbial behavior [1,2,3,4,7,8]. This may support local strategies aimed at reducing mucus stagnation, improving mechanical clearance, and disrupting biofilm architecture, including nasal irrigation, optimized topical therapy delivery, mucolytic approaches, and, in selected cases, microbiologically guided antimicrobial treatment. Cytological evidence of structured biofilm alone, however, should not be considered sufficient to justify antibiotics without clinical, endoscopic, and microbiological correlation.

Pattern IV, the inflammatory biofilm-associated pattern, may be the most relevant from a precision medicine perspective, since the therapeutic target is not only the microbial structure but the inflammatory microenvironment in which it is embedded. A neutrophil-rich pattern may suggest an infectious or innate inflammatory component, whereas association with eosinophils, mast cells, dense mucus, epithelial damage, or Charcot–Leyden crystals may indicate a more complex inflammatory phenotype. In chronic rhinosinusitis, especially with type 2 inflammation, treatment should not be reduced to an antimicrobial strategy: anti-inflammatory treatment, topical corticosteroids, control of eosinophilic and mast cell inflammation, restoration of epithelial barrier function, and management of comorbidities may be equally or more important than targeting the microbial component alone [9,10,11,12,18,19].

Pattern V, the dispersal/disrupted biofilm pattern, requires particularly careful interpretation, as fragmented material, bacterial satellites, and disrupted matrix-like structures may suggest active biofilm dispersion, mechanical disruption, treatment-related destabilization, or smear-preparation effects [5,6,7]. Its meaning depends strongly on the timing of sampling, including whether the sample was obtained before treatment, during therapy, after local cleansing, or during clinical worsening, and serial cytology may be especially useful for understanding whether a disrupted pattern reflects improvement, instability, or persistence of disease activity.

Recognizing mixed or composite patterns may further refine therapeutic interpretation: a dominant structured/mature biofilm pattern associated with neutrophilic inflammation suggests a different clinical scenario from a structured biofilm pattern associated with eosinophil–mast cell inflammation, and coexistence of structured biofilm with dispersal features may indicate partial biofilm disruption. Reporting the dominant pattern together with associated secondary patterns may help clinicians avoid simplistic therapeutic decisions based solely on the word “biofilm.”

Fungal elements require a separate level of therapeutic caution. Detection of isolated fungal spores should not be considered an indication for antifungal treatment, as spores may reflect environmental exposure, contamination, transient colonization, or saprophytic presence, whereas vegetative forms, especially septate or non-septate hyphae, require more careful evaluation. In uncomplicated paranasal sinus fungus ball, surgical removal is generally the main therapeutic approach, and systemic antifungal therapy is not routinely required in non-invasive cases [24]. By contrast, suspected invasive fungal rhinosinusitis, particularly in immunocompromised patients or those with poorly controlled diabetes, represents a potentially life-threatening condition requiring urgent multidisciplinary assessment, histopathological confirmation when possible, surgical debridement, and systemic antifungal therapy according to current recommendations [25,26].

Overall, the therapeutic implication of this classification is not that each cytological pattern corresponds to a specific treatment, but that each pattern may modify the level of clinical concern and the type of diagnostic–therapeutic reasoning: Pattern I may suggest caution and avoidance of overtreatment; Pattern II may support follow-up and optimization of local mucosal care; Pattern III may raise suspicion of persistent structured biofilm and encourage local antibiofilm-oriented strategies; Pattern IV may indicate the need to treat both microbial organization and inflammation; and Pattern V may require dynamic interpretation in relation to treatment timing, recurrence, or exacerbation. Fungal vegetative forms, especially hyphae, should prompt specific clinical correlation and, in high-risk patients, urgent evaluation.

In this sense, a cytological classification of nasal biofilms may support a more individualized approach to upper airway inflammation, helping avoid two opposite errors: overtreating harmless colonization and underestimating structured, inflammatory, mixed, or potentially invasive microbial patterns. Future studies should evaluate whether specific cytological biofilm patterns correlate with response to topical therapy, antibiotics, mucolytics, antibiofilm strategies, surgery, biologic treatment, or recurrence risk. Until such validation is available, the classification should be used as a tool for clinical interpretation and communication, not as an autonomous therapeutic decision system.

## 7. Limitations and Future Perspectives

The proposed cytological classification has several limitations that must be clearly acknowledged. Most importantly, it has not yet undergone prospective clinical validation and should therefore be regarded as a hypothesis-generating morphological framework rather than a validated diagnostic, prognostic, or therapeutic classification. The five proposed patterns are based on microscopic interpretation of bacterial organization, matrix-like material, inflammatory context, and possible dispersal features, but their reproducibility, biological correspondence, and clinical significance require formal prospective validation. Accordingly, the proposed categories should not presently be used as stand-alone criteria for diagnosis, prognosis, or treatment selection.

The anatomical compartment sampled by nasal cytology represents an additional limitation, because findings obtained from the inferior turbinate cannot be extrapolated automatically to the paranasal sinus mucosa. This distinction is particularly relevant in chronic rhinosinusitis and in suspected fungal sinus disease, where microbial composition and biofilm architecture may differ between the nasal cavity and the sinus environment.

Another limitation is that nasal cytology does not provide species-level microbial identification. The cytological detection of bacteria, fungal spores, hyphae, mixed microbial elements, or biofilm-like structures cannot determine whether the observed microorganisms are commensal, saprophytic, opportunistic, or pathogenic. Similarly, cytology cannot define virulence factors, antimicrobial resistance profiles, quorum-sensing activity, or the functional status of the microbial community. For this reason, cytological interpretation should always be integrated with clinical findings, endoscopy, microbiological culture, molecular methods, mycological assessment, and, when necessary, histopathology.

A further limitation concerns fungal findings. Nasal cytology may suggest the presence of fungal spores or vegetative fungal forms, including septate or non-septate hyphae, but it cannot establish tissue invasion. This distinction is particularly important in fungal rhinosinusitis, where non-invasive conditions such as fungus ball must be clearly distinguished from invasive fungal disease. Therefore, cytological evidence of hyphae, especially in immunocompromised patients or in patients with poorly controlled diabetes, should prompt urgent multidisciplinary correlation rather than being interpreted as a definitive diagnosis.

Pre-analytical and technical factors may influence the appearance of biofilm-like structures and represent an important limitation of cytological interpretation. Sampling, smear preparation, staining quality, and recent local or antimicrobial treatment may modify microbial aggregates and matrix-like material, particularly when evaluating disrupted or dispersal-like patterns. These potential sources of misinterpretation are discussed in detail in Section Differential Diagnosis and Diagnostic Pitfalls and reinforce the need for standardized sampling and slide preparation.

Observer experience represents another relevant issue. The distinction between free bacteria, microaggregates, structured biofilm-like formations, inflammatory biofilm-associated patterns, and disrupted biofilm-like material may require specific training in nasal cytology. Borderline categories may be particularly susceptible to observer variability, underscoring the need for formal reproducibility assessment, as detailed in the validation roadmap below.

### Proposed Roadmap for Validation

The proposed classification requires formal validation before it can be considered a reproducible diagnostic or prognostic system. A stepwise validation strategy should address analytical reproducibility, biological correspondence, external validity, and clinical relevance.

As a first step, a standardized image set representing the five proposed cytological patterns should be independently evaluated by multiple trained observers. Interobserver agreement should be quantified using appropriate kappa statistics, while repeated blinded assessment of the same images should be used to determine intraobserver reproducibility. Particular attention should be given to potentially overlapping categories, especially free bacterial colonization versus the microaggregate biofilm-like pattern and structured/mature versus dispersal/disrupted patterns.

The second step should assess biological correspondence between cytological morphology and established biofilm detection methods. Representative cytological patterns should be correlated, whenever feasible, with confocal laser scanning microscopy, scanning electron microscopy, fluorescence in situ hybridization (FISH), microbiological culture, and molecular approaches, including microbiome sequencing. Such multimodal comparison would help determine whether the morphological features identified by routine nasal cytology correspond to differences in microbial organization, composition, and biofilm architecture.

External validation should subsequently be performed in prospective multicenter cohorts using standardized sampling, staining, image acquisition, and reporting procedures. These studies should include different upper airway inflammatory conditions and evaluate the consistency of pattern recognition across centers, observers, and patient populations.

Finally, clinical validation should determine whether individual cytological patterns, or changes in pattern over time, correlate with symptoms, endoscopic findings, radiological disease burden, inflammatory phenotype, microbiological findings, treatment response, recurrence, and other clinically relevant outcomes. Longitudinal studies would be particularly valuable for establishing whether transitions among patterns represent true biological changes and whether the proposed classification provides information beyond conventional presence/absence assessment.

Until these levels of validation are achieved, the proposed classification should be regarded as a hypothesis-generating morphological framework intended to provide a standardized descriptive language and a basis for prospective investigation.

The integration of nasal cytology with microbiome analysis and digital pathology may also open new perspectives. Artificial intelligence-assisted image analysis could potentially support recognition of bacterial aggregates, matrix-like structures, inflammatory cell patterns, fungal elements, and dispersal morphology. However, before such tools can be clinically useful, large annotated datasets and standardized image acquisition protocols will be required [27].

Finally, the proposed classification should be viewed as an initial step toward a more precise language for describing nasal biofilms. Its main purpose is to stimulate discussion, standardize observation, and generate testable hypotheses. If validated, a pattern-based cytological approach may help move nasal biofilm assessment from a simple presence/absence report toward a more informative interpretation of microbial organization, host response, and disease behavior.

## 8. Conclusions

Nasal biofilms should not be interpreted solely as present or absent. This Perspective proposes a pattern-based cytological framework comprising five main patterns that reflect different degrees of microbial organization, host–microbe interaction, and possible biofilm dynamics. The proposed patterns are intended to provide a shared morphological language for nasal cytology and to support more structured interpretation of biofilm-related findings within their inflammatory and clinical context.

At present, this classification should be regarded as a hypothesis-generating framework rather than a validated diagnostic or prognostic system. Prospective multicenter studies assessing reproducibility, biological correspondence, and clinical relevance are required to determine whether this pattern-based approach can ultimately contribute to more precise phenotyping and personalized management of chronic and recurrent upper airway inflammation.

## Figures and Tables

**Table 1:** Summary of the proposed cytological classification of nasal biofilms.

Pattern	Standardized Morphological Criteria	Key Microscopic Features	Proposed Biological Interpretation	Clinical Note
**I—Free bacterial** **colonization**	Predominantly isolated or scattered cocci/bacilli; no cohesive bacterial cluster; no recognizable matrix-like background; bacteria mainly dispersed within mucus or superficial debris.	Scattered free bacteria (cocci/bacilli) within mucus; no matrix-like material or clustering	Possible transient colonization, contamination, or superficial overgrowth	Avoid automatic antimicrobial treatment; reassess if neutrophilic inflammation or symptoms are present
**II—Microaggregate** **biofilm-like pattern**	Small, focal bacterial clusters clearly distinguishable from scattered bacteria; loose or incomplete aggregation; absent or poorly defined matrix-like material; no compact three-dimensional architecture.	Small, loosely organized bacterial clusters; incomplete, poorly defined matrix	Early or unstable microbial organization	Favor follow-up and mucosal hygiene measures over immediate antimicrobial therapy
**III—Structured/mature** **biofilm pattern**	Cohesive, dense bacterial aggregate with compact architecture; bacteria embedded within recognizable amorphous/cyanophilic matrix-like material; irregular but well-defined aggregate borders; frequent association with mucus, epithelial material, or cellular debris.	Dense, compact aggregates embedded in cyanophilic matrix-like material (“infectious spot”)	Organized, mature biofilm	Consider mechanical/mucolytic clearance strategies; antibiotics only with clinical and microbiological correlation
**IV—Inflammatory** **biofilm-associated pattern**	Biofilm-like structure fulfilling Pattern II or III morphological features plus a conspicuous inflammatory-cell component surrounding or intermingled with the aggregate; inflammatory phenotype described qualitatively as neutrophilic, eosinophilic, mast-cell-associated, or mixed.	Biofilm-like structure embedded in neutrophilic, eosinophilic, or mast-cell infiltrate; possible Charcot–Leyden crystals	Active, clinically significant host–microbe interaction	Target the inflammatory component (e.g., topical corticosteroids) alongside the microbial component; distinguish infectious/innate from type 2 phenotype
**V—Dispersal/disrupted** **biofilm pattern**	Partial loss of compact architecture; fragmented matrix-like material; detached bacterial clusters or peripheral bacterial satellites around a residual aggregate; scattered bacteria adjacent to a disrupted central structure.	Fragmented aggregates, peripheral bacterial satellites, disrupted matrix-like material	Biofilm instability/dispersal, or treatment- and sampling-related artefact	Interpret in relation to timing of treatment and sampling; consider serial cytology
**Mixed/composite**	Two or more clearly recognizable patterns within the same smear; dominant and secondary pattern(s) reported separately.	Coexistence of ≥2 of the above patterns within the same smear	Spatial or temporal heterogeneity of biofilm organization	Report dominant and secondary patterns together with the prevailing inflammatory background

Note: No numerical thresholds for aggregate size, bacterial density, matrix extension, or inflammatory-cell counts are currently proposed, because such cut-offs have not yet been validated for nasal biofilm cytology. Their definition represents a specific objective of future reproducibility studies.

**Table 2:** Evidence supporting the proposed cytological biofilm patterns and distinction between published observations and expert interpretation.

Proposed Pattern	Published Evidence Supporting the Concept	Evidence Directly Available in Nasal Cytology	Interpretative Component of the Proposed Classification	Overall Evidence Status
**I. Free bacterial colonization**	Planktonic/free-living bacteria are biologically distinct from organized biofilm communities [1,2,3]. Nasal cytology permits direct visualization of free bacterial elements within mucus [13,14,15,16,17].	Scattered cocci/bacilli without compact aggregation or matrix-like material can be observed in routine nasal smears.	Interpretation as transient colonization, contamination, or superficial overgrowth depends on the clinical and inflammatory context.	**Moderate direct support**
**II. Microaggregate biofilm-like pattern**	Early biofilm development includes adhesion, bacterial clustering, microcolony formation, and progressive matrix production [4,5].	Small bacterial aggregates and intermediate forms are observable cytologically, although a specific validated nasal cytological category has not previously been established.	The interpretation of microaggregates as an early or unstable biofilm stage is primarily hypothesis-generating.	**Indirect/moderate support**
**III. Structured/mature biofilm pattern**	Mature biofilms are characterized by dense microbial organization and extracellular matrix production [1,2,3,4,7,8]. The “infectious spot” has been described in nasal cytology as a morphological-chromatic expression of biofilm [13,16,17], and biofilm-like structures have been reported in rhinitis and sinonasal disease [14,17]. Independent studies have further demonstrated and characterized sinonasal biofilms using complementary microscopic and molecular approaches [20,21,22].	Compact bacterial aggregates embedded in cyanophilic matrix-like material are directly recognizable in May–Grünwald–Giemsa preparations.	Classification of these findings specifically as the “structured/mature” category represents the proposed framework, although the underlying morphology is previously described.	**Strongest direct cytological support**
**IV. Inflammatory biofilm-associated pattern**	Biofilms and host inflammation coexist in chronic sinonasal disease [9,10,11,12,20,21,22,23]. Nasal cytology allows simultaneous assessment of microorganisms, epithelial cells, neutrophils, eosinophils, mast cells, mucus, and debris [15,18,19].	Biofilm-like structures may be visualized within neutrophilic, eosinophilic, mast-cell-rich, or mixed inflammatory backgrounds.	Subclassification according to the inflammatory context and its proposed clinical significance is based largely on expert interpretation and requires prospective validation.	**Moderate biological and cytological support**
**V. Dispersal/disrupted biofilm pattern**	Detachment and dispersion are recognized phases of the biofilm life cycle and may follow matrix degradation, environmental stress, or mechanical disruption [4,5,6,7].	Fragmented aggregates, residual matrix-like structures, peripheral bacterial satellites, and scattered bacteria can be observed cytologically.	Interpreting such morphology as biological dispersal rather than sampling, treatment, or smear artefact remains mainly hypothesis-generating.	**Indirect support; highest interpretative component**

## Data Availability

No new data were created or analyzed in this study. Data sharing is not applicable to this article.
